# Characteristic Constituents of Maocangzhu and Beicangzhu Revealed Using Electronic Nose, Electronic Tongue, HS-GC-IMS, and UPLC-Orbitrap Technologies

**DOI:** 10.3390/molecules31132350

**Published:** 2026-07-03

**Authors:** Hanqi Zhang, Zhenni Qu, Fan Wang, Yutong Han, Yanan Li

**Affiliations:** 1 Institute of Pharmacy (Institute of TCM Health Industrial Technology), Shandong University of Traditional Chinese Medicine, Jinan 250355, China; 2 Shandong Key Laboratory of Digital Traditional Chinese Medicine, Shandong University of Traditional Chinese Medicine, Jinan 250355, China

**Keywords:** Atractylodis Rhizoma, HS-GC-IMS, UPLC-Orbitrap MS, electronic nose, electronic tongue

## Abstract

Atractylodis Rhizoma is an important traditional Chinese medicinal material derived from two botanical origins, Maocangzhu (MCZ) and Beicangzhu (BCZ), which are difficult to distinguish by conventional morphological identification because of their similar appearance. However, differences in botanical origin may lead to variations in odor, taste, volatile constituents, and non-volatile metabolites, thereby affecting quality evaluation and clinical application. This study aimed to systematically characterize the sensory and chemical differences between MCZ and BCZ and to identify potential markers for their discrimination. A multi-dimensional analytical strategy combining electronic nose, electronic tongue, headspace gas chromatography–ion mobility spectrometry (HS-GC-IMS), and ultra-high-performance liquid chromatography–Orbitrap high-resolution mass spectrometry (UPLC-Orbitrap MS) was established. Electronic nose and electronic tongue were used to digitize odor and taste characteristics, HS-GC-IMS was employed to profile volatile organic compounds, and UPLC-Orbitrap MS was applied to characterize non-volatile metabolites. Principal component analysis (PCA), orthogonal partial least squares discriminant analysis (OPLS-DA), variable importance in projection (VIP) screening, permutation tests, and correlation analysis were further used to evaluate discrimination performance and screen characteristic markers. The electronic nose results showed that MCZ and BCZ exhibited distinct odor profiles, with W5S, W1W, and W1S identified as the main differential sensors, suggesting that nitrogen oxides, terpenoids, inorganic sulfides, and short-chain alkanes contributed to the odor differences between the two origins. Electronic tongue analysis further demonstrated clear taste discrimination, with sourness and richness identified as the key taste indicators. HS-GC-IMS detected 108 volatile organic compounds, and 24 volatile markers with VIP > 1.2 were screened as important contributors to the differentiation of MCZ and BCZ. Among them, propionic acid and 5-methyl-2-furancarboxaldehyde were mainly distributed in MCZ, whereas (E)-caryophyllene was present only or at higher levels in BCZ, indicating its potential as a characteristic volatile marker of BCZ. UPLC-Orbitrap MS detected 78 non-volatile constituents, and OPLS-DA screened 17 key non-volatile differential metabolites with VIP > 1.2. These results indicated that MCZ and BCZ could be clearly separated not only by sensory signals but also by volatile and non-volatile chemical profiles. This study revealed that the differences between MCZ and BCZ are mainly reflected in odor-active volatile compounds, key taste indicators, and non-volatile differential metabolites. The integration of electronic nose, electronic tongue, HS-GC-IMS, and UPLC-Orbitrap MS provides a comprehensive and reliable strategy for distinguishing the two botanical origins of Atractylodis Rhizoma. These findings provide valuable insights into the material basis underlying the sensory and chemical differences between MCZ and BCZ and offer scientific support for accurate authentication, quality evaluation, and rational clinical application of Atractylodis Rhizoma.

## 1. Introduction

Atractylodis Rhizoma is an important traditional Chinese medicinal material derived from the dried rhizomes of two Asteraceae species, *Atractylodes lancea* (Thunb.) DC. and *Atractylodes chinensis* (DC.) Koidz. In China, *A. lancea* is commonly known as Maocangzhu and is abbreviated as MCZ, whereas *A. chinensis* is commonly known as Beicangzhu and is abbreviated as BCZ. MCZ, also known as Nancangzhu, is mainly distributed in Jiangsu, Hubei, and Henan provinces of China, with the Maoshan region in Jiangsu Province traditionally recognized as an authentic production area. BCZ, also known as Shancangzhu, is primarily produced in Jilin, Liaoning, Inner Mongolia and Hebei provinces of China [1]. According to traditional Chinese medicine theory, Atractylodis Rhizoma has the effects of drying dampness, strengthening the spleen, dispelling wind, relieving cold, and improving vision. Modern pharmacological studies have reported that Atractylodis Rhizoma possesses multiple bioactivities, including antitumor [2], antibacterial [3], anti-inflammatory [4], and immunomodulatory effects [5].

Although MCZ and BCZ are both botanical origins of Atractylodis Rhizoma, their chemical compositions may differ because of genetic background, geographical distribution, and growth environment [6]. However, the two herbal materials are highly similar in external morphology, and conventional identification methods based mainly on appearance, odor, and empirical experience are often insufficient for accurate discrimination. This may lead to misidentification or mixed use in herbal markets and clinical practice, thereby affecting quality consistency and clinical efficacy. Therefore, it is necessary to establish reliable analytical methods for distinguishing MCZ from BCZ and to clarify the characteristic constituents responsible for their differences.

Atractylodis Rhizoma has a complex chemical profile, containing volatile constituents such as essential oils and terpenoids, as well as non-volatile constituents such as sesquiterpenes, polysaccharides, phenolic acids, and other secondary metabolites. Conventional analytical methods, such as gas chromatography–mass spectrometry (GC-MS), can effectively characterize essential oils [7]. However, these methods usually require relatively complex sample preparation and longer analysis time, which may limit their suitability for high-throughput rapid screening. Headspace gas chromatography–ion mobility spectrometry (HS-GC-IMS) combines chromatographic separation with ion mobility detection, enabling relatively rapid analysis of volatile organic compounds (VOCs) with simplified sample pretreatment. It is particularly suitable for detecting small-molecule VOCs and has been increasingly applied in the analysis of traditional Chinese medicines [8].

In addition to chemical profiling, sensory characteristics such as odor and taste are important features traditionally used in the identification and quality evaluation of Chinese medicinal materials. Electronic nose and electronic tongue technologies provide objective, reproducible, and digitalized evaluation of odor and taste, helping reduce the subjectivity associated with manual sensory assessment. These biomimetic techniques have shown considerable potential in quality control and authenticity evaluation of traditional Chinese medicines [9]. Meanwhile, Orbitrap mass spectrometry, characterized by high resolution, broad spectral coverage, and excellent mass accuracy, enables reliable multistage mass spectrometric analysis of complex samples and has been widely used for chemical component characterization [10,11].

Given the complexity of Atractylodis Rhizoma, a single analytical technique is insufficient to comprehensively reveal the differences between MCZ and BCZ. Therefore, the aim of this study was to systematically compare the two botanical origins by integrating electronic nose, electronic tongue, HS-GC-IMS, and UPLC-Orbitrap MS from sensory, volatile, and non-volatile perspectives, and to identify the characteristic constituents distinguishing MCZ from BCZ, thereby providing a scientific basis for the accurate authentication, quality evaluation, and rational utilization of Atractylodis Rhizoma.

## 2. Results

### 2.1. HS-GC-IMS Analysis of Volatile Metabolites

#### 2.1.1. Identification of Major Volatile Metabolites in *Atractylodes* Samples Based on HS-GC-IMS

**Figure 1:**
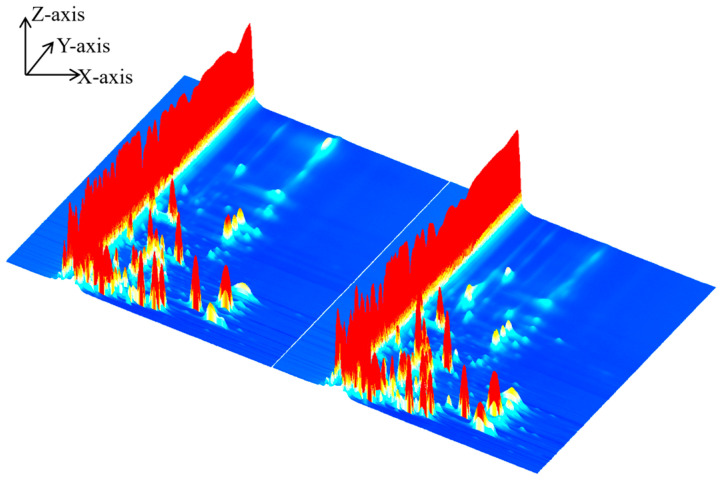
3D gas-phase ion mobility spectrum comparison.

**Figure 2:**
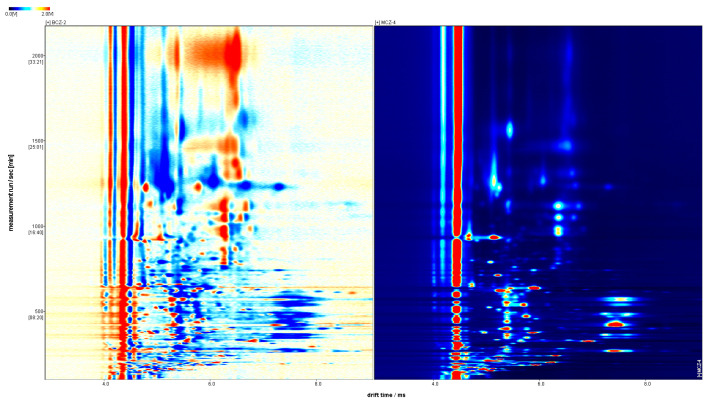
Differential comparison spectra.

Figure 1 presents the three-dimensional (3D) surface characteristics of volatile metabolites identified in Atractylodis Rhizoma samples from different sources. The X, Y, and Z axes of the 3D plot correspond to migration time, retention time, and peak intensity, respectively. The 3D plots reveal pronounced differences in the types and concentrations of volatile organic compounds between BCZ and MCZ. For clearer comparison among samples, a differential comparison mode was used: each point represents a specific volatile organic compound, and the color intensity reflects concentration, with darker shades indicating higher concentrations. The spectrum of BCZ was set as the reference, with the spectrum of MCZ subtracted from it. Red signifies a higher concentration than the reference, while blue signifies a lower concentration. The results from the differential comparison spectrum (Figure 2) confirm differences in both component types and concentrations between MCZ and BCZ, consistent with the observations from the 3D spectrum.

Following background subtraction, chromatographic peak extraction, and peak alignment of the GC-IMS data from MCZ and BCZ samples, a total of 108 active components were identified and qualitatively analyzed (Table 1). These components primarily include esters, aldehydes, ketones, alcohols, and acids. Specifically, there were 13 aldehydes (12.04%), 14 ketones (12.96%), 21 alcohols (19.44%), 23 esters (21.30%), 4 carboxylic acids (3.70%), 8 phenols (7.41%), 6 alkenes (5.56%), 7 nitrogen-containing compounds (6.48%), 4 sulfur-containing compounds (3.70%), and 8 other compounds (7.41%). Some of these compounds are present as both monomers and dimers.

#### 2.1.2. Analysis of Major Volatile Metabolites in *Atractylodes* Samples Using HS-GC-IMS

**Figure 3:**
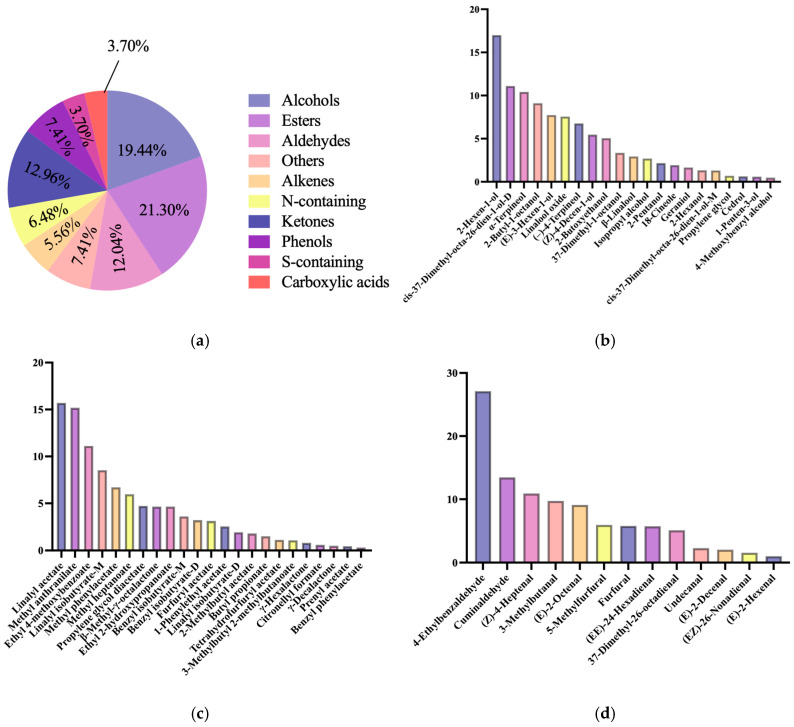
Composition of major volatile metabolites. (**a**) Pie chart of relative volatile metabolite content; (**b**) alcohols; (**c**) esters; (**d**) aldehydes.

2-Hexen-1-ol, geraniol, and α-Terpinolene are major alcohol-type volatile metabolites in *Atractylodes* (Figure 3b). 2-Hexen-1-ol is a nearly colorless liquid with a strong odor reminiscent of unripe fruit. Studies have shown that 2-hexen-1-ol exhibits inhibitory effects against Fusarium species infecting stored corn grains [12]. Geraniol, a monoterpenol with a rose-like fragrance, is widely used in cosmetics and high-end perfumes; it also demonstrates antibacterial [13], anticancer [14], neuroprotective [15,16], and antioxidant activities. α-Terpineol is a monocyclic monoterpene alcohol commonly found in the essential oils of various aromatic plants. It has been reported to possess a broad spectrum of biological activities, including anticancer, anti-inflammatory, and analgesic properties. α-Terpineol has been demonstrated to exert anticancer effects by suppressing NF-κB signaling and inducing apoptosis in tumor cells [17]. Moreover, its antinociceptive and anti-inflammatory effects have been validated in neuropathic pain models, where it significantly attenuated mechanical allodynia and hyperalgesia through the inhibition of spinal microglial activation and inflammatory cytokines [18].

Other bioactive compounds have also been identified among the volatile alcohol metabolites of *Atractylodes*, contributing to its complex and unique chemical profile and biological functions.

Linalyl acetate, Methyl anthranilate, ethyl 4-methoxybenzoate, and Linalyl isobutyrate together accounted for 50.6% of all ester compounds detected in this study (Figure 3c). Linalyl acetate is a monoterpene compound and the acetate ester of linalool, constituting a major component of bergamot and lavender essential oils. Its biological activity is closely related to that of linalool, and it may help alleviate psoriasiform inflammation by interfering with IL-12-driven inflammatory responses, mainly acting on Th-1 cell-specific cytokines [19]. Methyl anthranilate may inhibit the quorum sensing system in Pseudomonas aeruginosa by interfering with AHL biosynthesis and competitively binding to receptor proteins. It shows promise as a quorum sensing inhibitor and anti-biofilm agent, with potential for improving food safety [20]. Ethyl 4-methoxybenzoate is a colorless, oily liquid with a sweet, anise-like aroma. Occurring naturally in apple brandy, plums, and white wine, it is primarily used as a fragrance ingredient in cosmetics and perfumes. Safety evaluations indicate it poses no health risks, such as genotoxicity or skin sensitization, at current usage levels and is safe for the aquatic environment [21]. Linalyl isobutyrate is a colorless to pale yellow liquid with a sweet, fresh, rose-like scent, widely used as a fragrance ingredient in perfumes, cosmetics, and personal care products [22].

4-ethylbenzaldehyde and cuminaldehyde are primary aldehyde components. 4-ethylbenzaldehyde is a colorless to pale yellow liquid with a sweet, bitter almond scent. It occurs naturally in various foods such as prickly ash, dairy products, and peanuts, and is used as a fragrance ingredient in products like lip care items, perfumes, and air fresheners [23]. Cuminaldehyde, also a colorless to pale yellow liquid, has a strong, pungent, spicy, green herbal odor. As a bioactive component of essential oils, cuminaldehyde shows potential for analgesic, anti-inflammatory, and antibacterial applications [24,25,26]. Cuminaldehyde exerts analgesic and neuroprotective effects via opioid receptors, the L-arginine/NO/cGMP/KATP signaling pathway, and anti-inflammatory functions. It can modulate immune responses in inflammation and may be considered a lead compound for novel anti-inflammatory and analgesic drug development [24,25].

#### 2.1.3. HS-GC-IMS-Based Volatile Organic Compound Fingerprint Analysis of *Atractylodes* Samples

**Figure 4:**
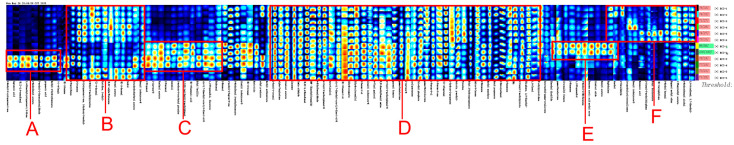
Component fingerprint profiles.

To further examine the differences between BCZ and MCZ, the Gallery Plot plugin was employed for a visual comparison of volatile organic compounds among various samples. All peaks were included for fingerprint analysis; in the figure, each row corresponds to all signal peaks from a single sample, and each column represents the signal peaks of the same volatile organic compound across different samples. Fingerprint spectra were constructed for BCZ and MCZ, as shown in Figure 4. A deeper red indicates a higher concentration of that component. The samples display both unique characteristic signals and shared common signals.

As illustrated in Figure 4, the components in regions A, C, and E are mainly concentrated in MCZ samples, including propionic acid and 5-methyl-2-furancarboxaldehyde, which may serve as volatile markers to differentiate BCZ from MCZ. The volatile profiles of batches 3 and 4 of BCZ are relatively similar in region A, while region E highlights the similarity between batches 1 and 2. Components in region B are present at higher levels in BCZ compared to MCZ. Region D includes 42 compounds common to both species. Region F comprises components found exclusively or at higher concentrations in MCZ; among these, (E)-Caryophyllene is unique to BCZ, consistent with previous literature [27], and can serve as a key marker for distinguishing between the two species.

#### 2.1.4. Multivariate Statistical Analysis Based on HS-GC-IMS

**Figure 5:**
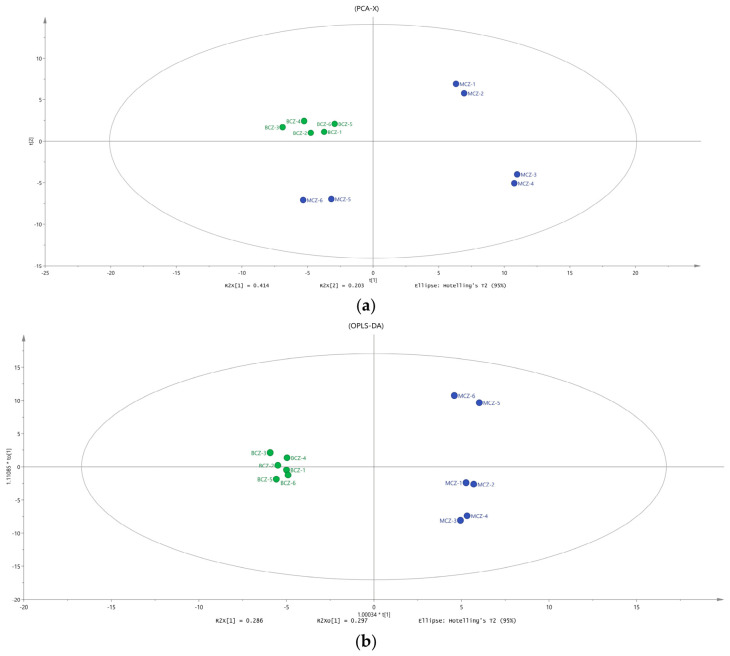

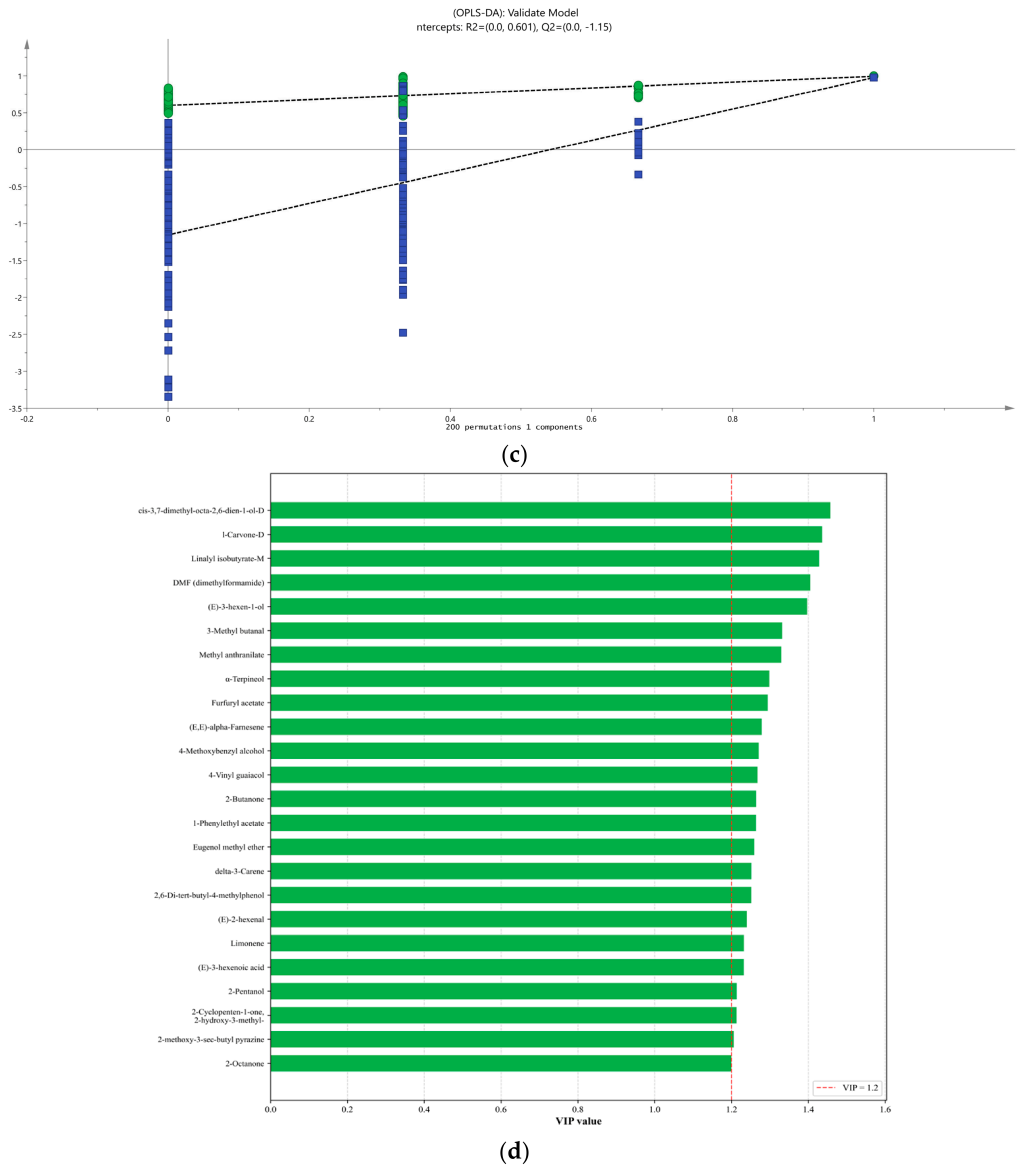
PCA and OPLS-DA based on HS-GC-IMS data. (**a**) PCA score plot; (**b**) OPLS-DA score plot; (**c**) permutation test; (**d**) VIP values.

The PCA scatter plot (see Figure 5) shows no clear separation between MCZ and BCZ samples, indicating that the data (variables and samples) used in this analysis were insufficient for PCA to clearly demonstrate the differences between these two categories. In contrast, the OPLS-DA scatter plot demonstrates a clear separation trend, suggesting that this model can reliably differentiate between MCZ and BCZ. Model assessment revealed that the cumulative explanatory power of independent variables (R^2^X) was 0.749, the goodness-of-fit for dependent variables (R^2^Y) was 0.994, and the predictive capability parameter (Q^2^) was 0.977. Both R^2^ and Q^2^ values are close to 1, reflecting excellent model fit and predictive reliability. The model’s robustness was further confirmed by a 200-permutation test, yielding a Q^2^ intercept of −1.15 (less than 0), indicating the model is not overfitted and remains stable and valid.

Using a VIP > 1.2 criterion, 24 volatile biomarkers were identified as significant contributors to differentiation between the two sample types, including: FCC (dimer), carvone (dimer), linalyl isobutyrate (monomer), dimethylformamide (DMF), (E)-3-hexen-1-ol, 3-methylbutyraldehyde, methyl anthranilate, α-terpineol, acetyl furfuryl ester, (E,E)-α-Farnesene, 4-methoxybenzyl alcohol, 4-vinylguaiacol, 2-butanone, 1-phenylethyl acetate, eugenol methyl ether, 3-δ-carene, 2,6-di-tert-butyl-4-methylphenol (monomer), (E)-2-hexenal, (E)-Caryophyllene, (E)-3-hexenoic acid, 2-pentanol, 2-hydroxy-3-methyl-2-cyclopent-1-enone, 2-methoxy-3-sec-butylpyrazine, and 2-Octanone.

### 2.2. UPLC-Orbitrap Analysis of Non-Volatile Metabolites

#### 2.2.1. Identification of Major Non-Volatile Metabolites in *Atractylodes* Samples Using UPLC-Orbitrap

**Figure 6:**
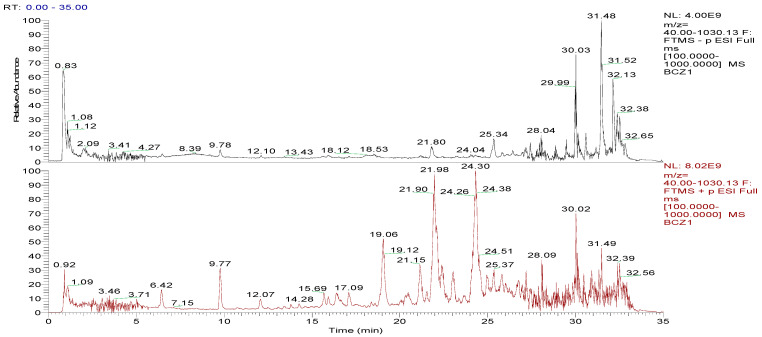
Total ion current chromatogram of non-volatile metabolites.

High-resolution mass spectrometry (HRMS) analysis was conducted using ESI, alternating between positive and negative ion modes, with a full MS/ddMS2 scan mode (Figure 6). Compound identification was achieved by comparing characteristic ion fragmentation patterns against in-house databases (mzCloud and mzVault), the PubChem online database (https://pubchem.ncbi.nlm.nih.gov/), and literature data. A total of 78 chemical constituents were identified (Table 2), including terpenes (15), organic acids and their esters (38), coumarins (4), lignans (2), flavonoids (2), sugars and glycosides (8), amides (5), and other components (4).

#### 2.2.2. Analysis of Major Non-Volatile Metabolites in *Atractylodes* Samples Using UPLC-Orbitrap

Terpenoids are the signature metabolites of *Atractylodes*. Atractylone is a characteristic compound of *Atractylodes* from authentic production areas, with demonstrated antitumor and anti-inflammatory properties [28,29]. Zedoarondiol has demonstrated anti-inflammatory effects [30] and anti-atherosclerotic activity [31]. Caryophyllene oxide exhibits multiple pharmacological activities, including anti-inflammatory, antioxidant, and antitumor effects [32,33,34,35]. Nootkatone (NKT) displays a range of biological activities, such as anti-inflammatory, anticancer, antibacterial, and neuroprotective effects [36,37,38,39,40].

Organic acids and their esters: Quinic acid demonstrates a variety of pharmacological activities, including antioxidant, antibacterial, anti-inflammatory, antiviral, and antitumor effects [41,42]. Chlorogenic acid also exhibits significant biological activities, such as anti-inflammatory, immunomodulatory, glucose- and lipid-lowering, antibacterial, and antitumor effects [43,44,45,46,47], making it highly applicable in medicine, healthcare, food, cosmetics, and as an alternative to antibiotics in animal feed. Arginine displays immunomodulatory, antioxidant, and lipid-lowering properties, as well as anti-inflammatory effects [48,49,50].

Coumarins and flavonoids: The coumarin compounds scopoletin and scoparone possess anticoagulant and vasoprotective effects [51,52,53,54]. The flavonoids daidzein and puerarin are notable for their antioxidant and anti-inflammatory properties, garnering significant scientific interest [55,56].

The active metabolites of *Atractylodes* are mainly terpenoids (zedoarondiol, atractylone, and NKT), alongside phenolic acids (chlorogenic acid, caffeic acid), coumarins (scopoletin, scoparone), and flavonoid glycosides (puerarin). Together, these compounds form the pharmacological basis for the herb’s traditional effects of “strengthening the spleen, drying dampness, and providing anti-inflammatory and hepatoprotective actions”. These results are highly consistent with modern pharmacological interpretations of *Atractylodes*’ traditional uses [57], supporting its application in metabolic and digestive system disorders.

#### 2.2.3. Multivariate Statistical Analysis Based on UPLC-Orbitrap

**Figure 7:**
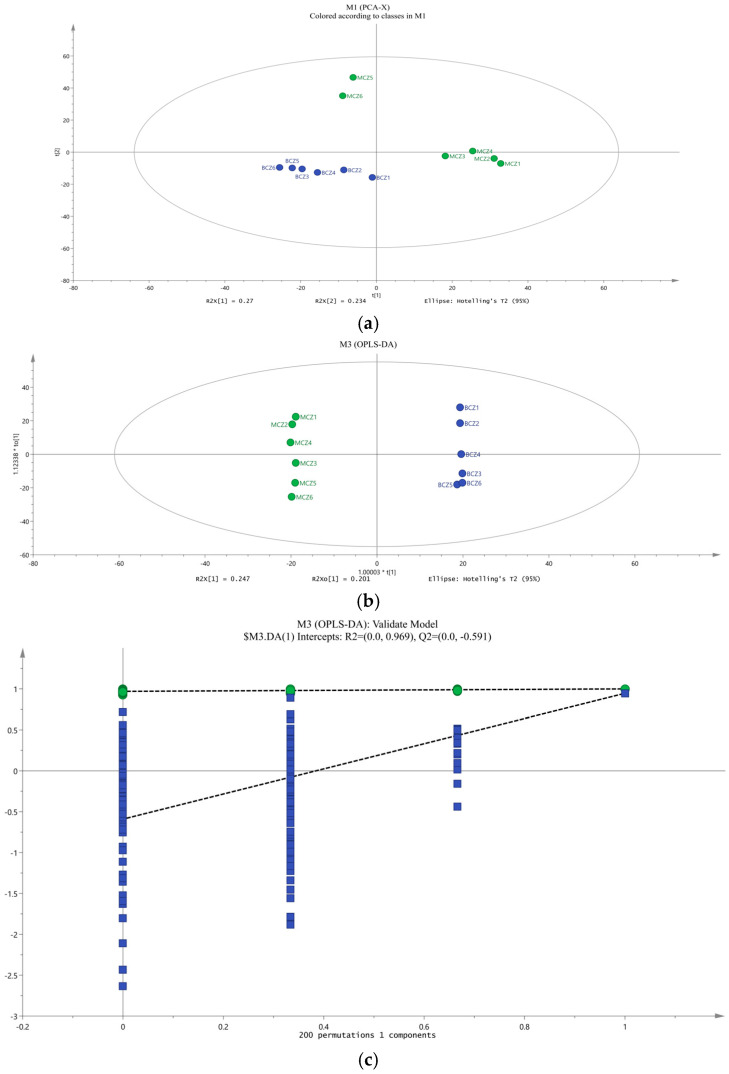

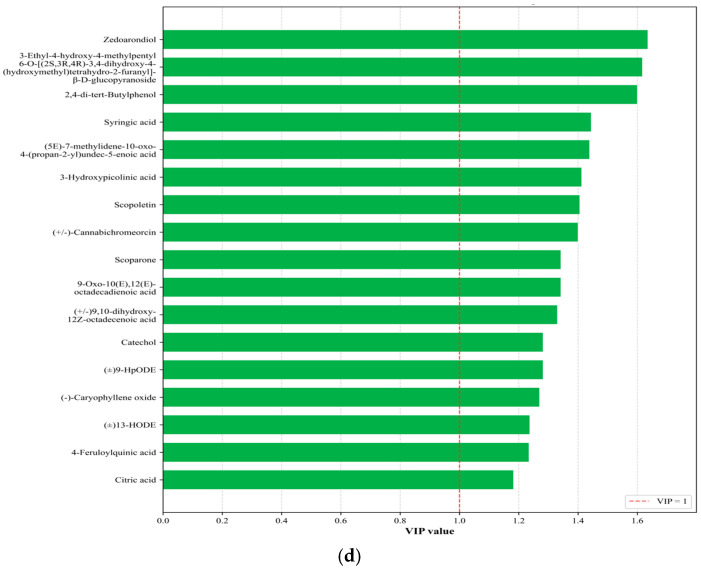
PCA and OPLS-DA based on UPLC-Orbitrap data. (**a**) PCA score plot; (**b**) OPLS-DA score plot; (**c**) Permutation test; (**d**) VIP values.

As shown in Figure 7a, although the PCA score plot can preliminarily distinguish between the two types of *Atractylodes* samples, its cumulative explanatory power is only 50.4%, indicating that PCA alone has limited ability to differentiate between varieties. In contrast, the OPLS-DA score plot in Figure 7b shows a strict separation boundary along the predicted component axis (t[1]): all BCZ samples (BCZ1–BCZ6) are distributed on the right, while all MCZ samples (MCZ1–MCZ6) are on the left, with no overlap, visually confirming the model’s high discriminatory capability. The OPLS-DA model (M3) explains 64.9% of the variance in the original data (R^2^X = 0.649), achieves a near-perfect fit for the classification labels (R^2^Y = 0.999), and its predictive power (Q^2^ = 0.946) markedly surpasses that of PCA (Q^2^ = 0.214) and PLS-DA (Q^2^ = 0.870). After a 200-permutation test, the R^2^ regression line intercept (0.969) was much lower than the original, and the Q^2^ regression line intercept (−0.591) was below zero, confirming that the model is valid and not overfitted as Figure 7c.

In this study, an OPLS-DA model was established to distinguish *Atractylodes* varieties based on non-volatile metabolites. The model’s performance was systematically validated through key statistical parameters and visualization. Furthermore, 17 non-volatile metabolites were identified as significant differentiators using UPLC-Orbitrap data with a VIP > 1.2 criterion as Figure 7d.

### 2.3. Electronic Nose Analysis Results

#### 2.3.1. Odor Composition Analysis of Northern *Atractylodes* and Maocang *Atractylodes*

**Figure 8:**
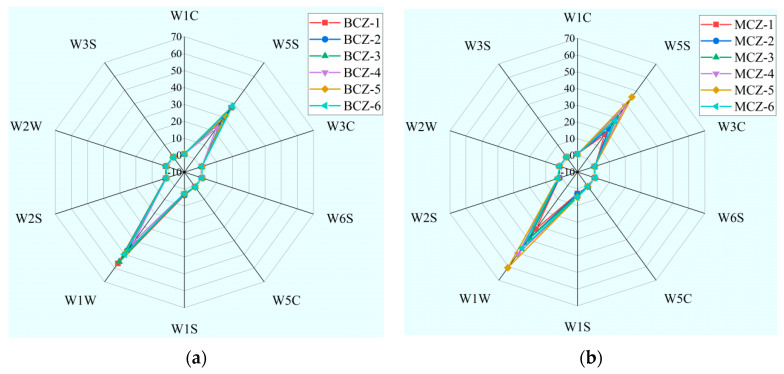
Radar plots of *Atractylodes* sensor responses. (**a**) Radar plot of MCZ sensor responses; (**b**) radar plot of BCZ sensor responses.

The average response values of electronic nose sensors for MCZ and BCZ samples were analyzed, and a radar chart was generated to visually illustrate the differences between the samples. As shown in Figure 8, all 10 sensors responded to the samples, with sensors W5S and W1W showing particularly strong responses. This suggests that both MCZ and BCZ contain higher levels of nitrogen oxides, terpenes, and inorganic sulfides, which are likely the main contributors to the odor differences between these species.

#### 2.3.2. Analysis of Contribution Rates to Sensor Discrimination via Loadings

**Figure 9:**
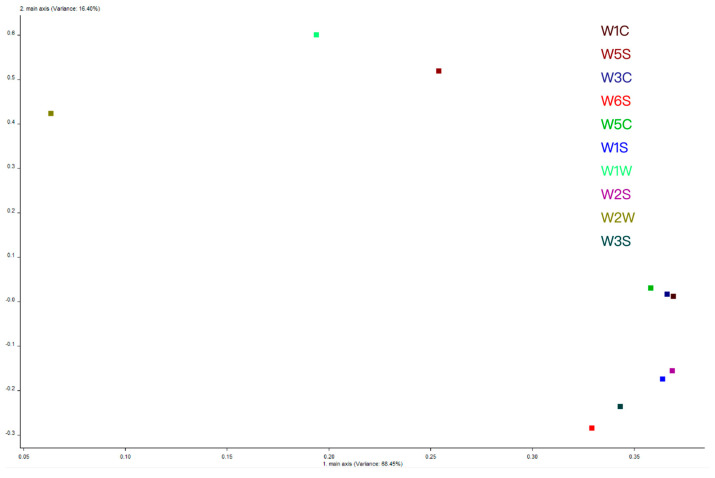
Analysis of sensor discrimination contribution rates.

Loadings analysis distinguishes the volatile components that play a primary role in each sample. As shown in Figure 9, the contribution rate of sensor response values for PC1 is 68.45% and for PC2 is 16.40%, with a cumulative contribution rate of 84.85%. Among these, W5S (Sensor No. 2) contributed most to discriminating the first principal component, while W1W (Sensor No. 7) contributed most to the second principal component. Based on the response characteristics, the first principal component is mainly sensitive to nitrogen oxides, and the second principal component to terpenes and inorganic sulfides. These findings are consistent with the earlier sensor response analysis.

#### 2.3.3. Multivariate Statistical Analysis Based on the Electronic Nose

**Figure 10:**
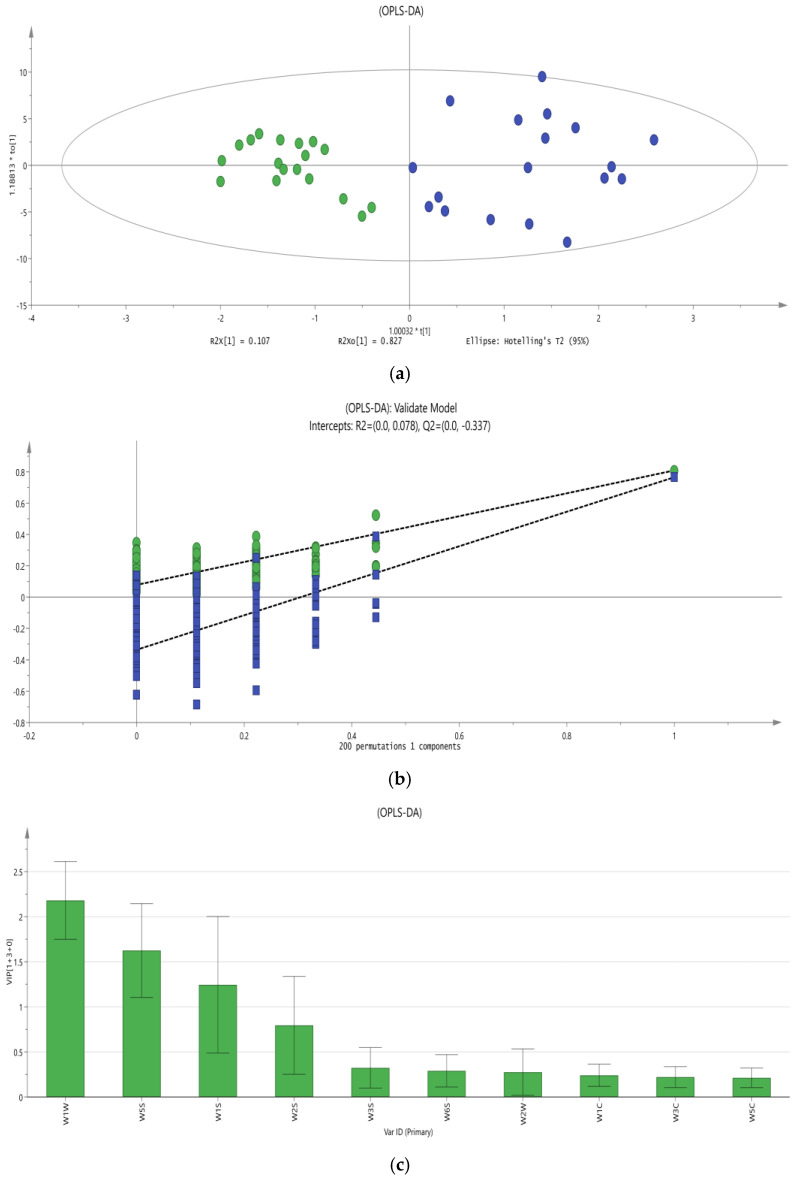
OPLS-DA based on electronic nose data. (**a**) OPLS-DA score plot; (**b**) permutation test; (**c**) VIP values.

Multivariate statistical analysis was performed using SIMCA 14.1 software for OPLS-DA analysis, with VIP > 1 as the screening criterion. Additionally, 200 bootstrap tests were conducted on the OPLS-DA models. As shown in Figure 10, after bootstrapping, the R^2^ and Q^2^ values on the right were higher than the original points on the left, indicating that the models did not suffer from overfitting and could reliably discriminate between BCZ and MCZ. The results show that the two species cluster into distinct groups: MCZ is mainly distributed in the first and fourth quadrants, and BCZ in the first and third quadrants. Using VIP > 1, three differentially responsive sensors (W1W, W5S, W1S) were identified.

Analysis of the electronic nose data indicates significant differences in odor characteristics between BCZ and MCZ, mainly reflected in the responses of sensors W5S, W1W, and W1S. Nitrogen oxides, terpenes, inorganic sulfides, and short-chain alkanes such as methane are the key volatile components distinguishing the two species.

### 2.4. Electronic Tongue Analysis Results

#### 2.4.1. Analysis of Taste Composition of BCZ and MCZ

**Figure 11:**
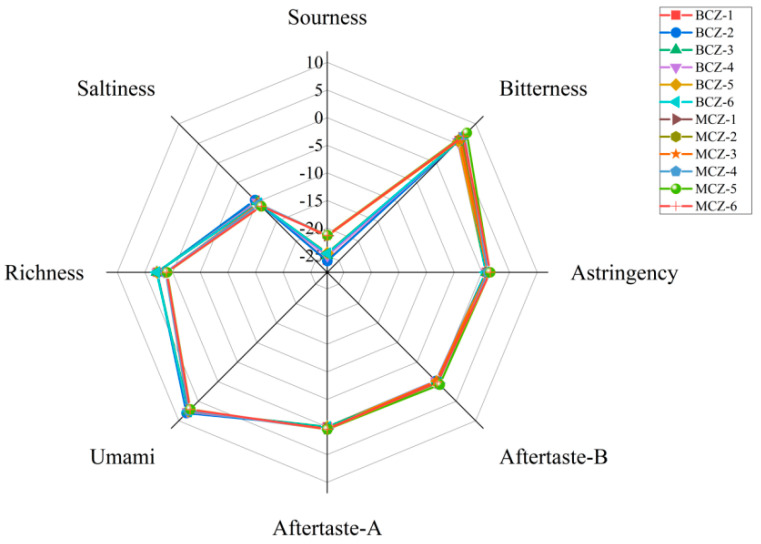
Electronic tongue sensor response values.

Figure 11 presents the radar chart of electronic tongue sensor response intensities to BCZ and MCZ samples. The results indicate that neither sample exhibits sourness, but both show relatively high umami and bitterness values. This aligns with the description of *Atractylodes* as “bitter” in the 2025 edition of the Chinese Pharmacopoeia.

#### 2.4.2. Multivariate Statistical Analysis Based on the Electronic Tongue

**Figure 12:**
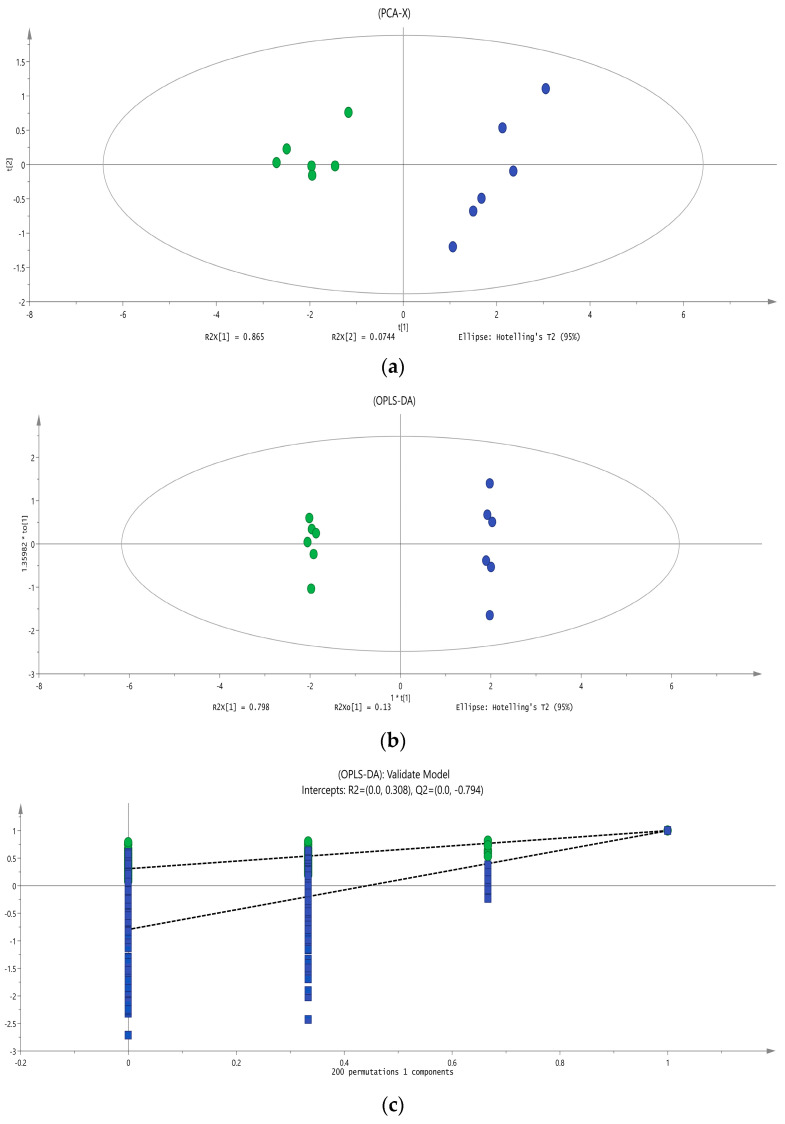

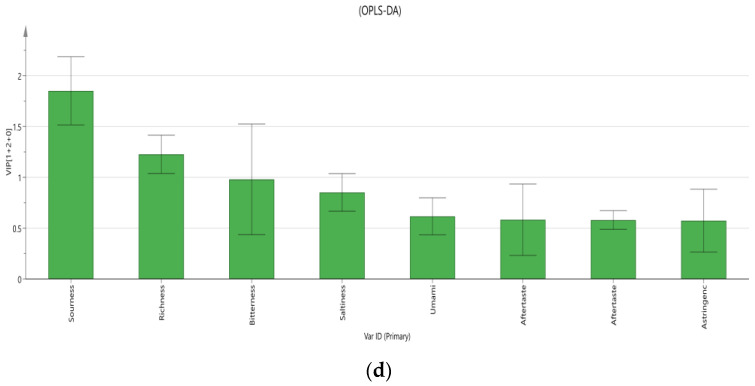
PCA and OPLS-DA based on electronic tongue data. (**a**) PCA score plot; (**b**) OPLS-DA score plot; (**c**) permutation test; (**d**) VIP values.

The electronic tongue sensor response values for BCZ and MCZ were analyzed using SIMCA 14.1 software for PCA. The results (Figure 12) show that the two species can be clustered into separate groups, indicating that the electronic tongue can distinguish *Atractylodes* from different origins.

To further identify distinguishing features between MCZ and BCZ, OPLS-DA analysis was performed on electronic tongue response values using SIMCA 14.1 software. With VIP > 1 as the criterion and 200 bootstrap tests, the results (Figure 12) show that the model is stable, reliable, and has strong predictive power without overfitting. The OPLS-DA plot shows that different *Atractylodes* species cluster into distinct groups, consistent with PCA results. Two discriminant features—sourness and richness—were identified. It should be noted that although the absolute sourness sensor response values were low for both BCZ and MCZ (Figure 11), the sourness sensor still generates a measurable electrical response, and the subtle difference in this response between MCZ and BCZ was statistically significant, which led to its identification as a discriminant feature by OPLS-DA (VIP > 1). In other words, electronic tongue sensors detect electrochemical signals rather than human taste perception; a statistically significant sensor difference does not necessarily indicate a perceptible taste difference at the human sensory threshold. Nevertheless, these subtle sensor-level differences, when combined with other taste attributes through multivariate modeling, contribute to the overall discrimination between the two species.

This study analyzed the flavor data of MCZ and BCZ and performed PCA using characteristic indicators. The results showed that the two species can be reliably distinguished by origin. OPLS-DA analysis further confirmed that flavor characteristics play a significant role in distinguishing *Atractylodes* origins. These findings provide a reference for origin identification and quality evaluation of *Atractylodes*.

### 2.5. Pearson Correlation Analysis of “Composition-Odor”

To further investigate the relationship between volatile metabolites and electronic nose sensor responses, a correlation analysis was conducted between major differentially expressed volatile metabolites and the sensors showing strong responses (W1W, W5S, and W1S). Twenty-four volatile metabolites with VIP > 1.2 were selected for correlation analysis with these three sensors.

**Figure 13:**
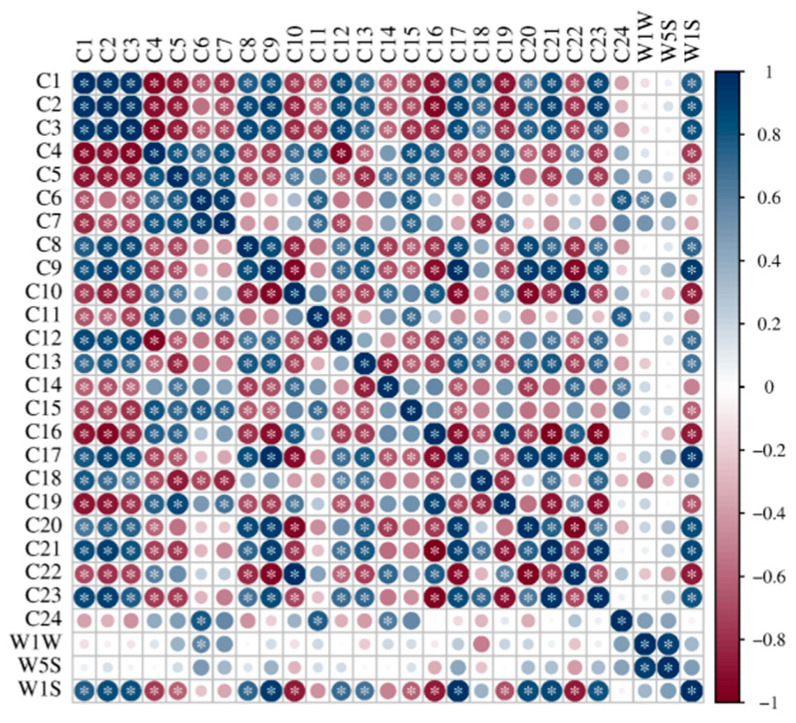
Correlation analysis plot (* *p* < 0.05).

The results (Figure 13) show that sensor W1W has a significant positive correlation with 3-methylbutyraldehyde (C6) (*p* < 0.05). Although W1W is sensitive to terpenes and inorganic sulfides, 3-methylbutyraldehyde—a short-chain aldehyde—may have produced a cross-response due to its molecular structure or sensor-surface interactions. No significant correlation was found between sensor W5S and any component (*p* > 0.05). Sensor W1S showed significant positive correlations with linalool (C1), carvone (C2), 4-vinylguaiacol (C12), 2-butanone (C13), 2,6-di-tert-butyl-p-cresol (C17), 2-pentanol (C21), and 2-methoxy-3-sec-butylpyrazine (C23), and significant negative correlations with farnesene (C10), δ-3-carene (C16), and 3-cyclopentene-1-one (C22).

Based on these findings, the main chemical basis for odor differences among the samples is attributed to short-chain oxygen-containing compounds (such as alcohols, ketones, and aldehydes) and terpenoids. Linalool, carvone, 4-vinylguaiacol, and 2-methoxy-3-sec-butylpyrazine produce strong positive responses on sensor W1S and are key contributors to the characteristic odors of the samples, while terpenes like farnesene and δ-3-carene may suppress or modify overall odor. Sensor W1S responds to the majority of differential components, making it the most critical sensor for distinguishing the odor characteristics of the samples.

### 2.6. Double Cross-Validation of OPLS-DA Models

Double cross-validation (2CV) was performed to rigorously evaluate the predictive ability of each OPLS-DA model. In Table 3, the 2CV Q^2^ values confirmed the robust predictive ability of all four OPLS-DA models, with 2CV Q^2^ of 0.624, 0.996, 0.880, and 0.878 for the E-nose, E-tongue, HS-GC-IMS, and UPLC-Orbitrap MS models, respectively. The classification accuracies ranged from 91.67% to 100%. All permutation test Q^2^ intercepts were negative, confirming that none of the models suffered from overfitting.

## 3. Discussion

This study integrated electronic nose, electronic tongue, HS-GC-IMS, and UPLC-Orbitrap MS to systematically analyze the sensory characteristics and chemical compositions of MCZ and BCZ. The results revealed significant differences between the two botanical origins in odor, taste, volatile compounds, and non-volatile metabolites.

HS-GC-IMS identified 108 volatile organic compounds, with 24 key volatile markers screened out, which contributed significantly to the differentiation of MCZ and BCZ. Notably, propionic acid and 5-methyl-2-furancarboxaldehyde were predominantly found in MCZ, while (E)-caryophyllene was enriched in BCZ, suggesting these compounds as potential volatile markers for distinguishing the two botanical origins. Ma et al. employed GC-MS coupled with chemometric analysis to differentiate four *Atractylodes* species, identifying 50 volatile components from 59 batches of samples and screening out hinesol, β-eudesmol, atractylon, atractylodin, and atractylenolide I as species characteristic markers [7]. However, traditional GC-MS analysis typically relies on hydrodistillation, which may cause loss or transformation of thermally labile compounds. In contrast, HS-GC-IMS requires no complex sample pretreatment and can more authentically reflect the original volatile profile. This technical advantage is particularly important for distinguishing botanical origins with subtle differences in volatile composition. Compared with conventional GC-MS, HS-GC-IMS offers advantages such as rapid analysis, minimal sample pretreatment, and high sensitivity for volatile organic compounds; however, its compound library is relatively limited compared to the well-established NIST mass spectral database used in GC-MS [58,59]. Therefore, combining both techniques provides a more comprehensive approach for *Atractylodes* quality evaluation.

Furthermore, UPLC-Orbitrap MS detected 78 non-volatile constituents, with 17 differential metabolites screened out by OPLS-DA with VIP > 1.2. Li et al. applied UHPLC-Orbitrap-HRMS for non-targeted metabolomics analysis of A. chinensis from eight origins, confirming significant metabolic variations among different regions [60]. These findings indicate that MCZ and BCZ can be effectively discriminated based on both sensory signals and chemical profiles, including volatile and non-volatile constituents.

Sensory technologies are also widely used in botanical origin identification. Wang et al. established a discrimination model for Codonopsis Radix from different origins by data fusion of electronic nose and electronic tongue, effectively addressing the issue of imbalanced sample categories [61], which confirmed the practical value of sensory technologies in botanical origin identification. This study found that the odor differences between MCZ and BCZ were mainly captured by the W5S, W1W, and W1S sensors, suggesting that nitrogen oxides, terpenoids, inorganic sulfides, and short-chain alkanes play key roles in the formation of distinct odor profiles of the two origins. Further electronic tongue analysis indicated that sourness and richness were the key taste indicators for distinguishing MCZ from BCZ. This finding underscores the value of electronic tongue-based digital taste profiling as a complementary tool to conventional chemical analysis, as it can detect subtle sensor-level differences that may not correspond to human sensory perception but nonetheless contribute to multivariate discrimination. Moreover, correlation analysis revealed that the response of the W1S sensor was strongly correlated with several key volatile compounds such as linalool and carvone. This analysis further confirmed that short-chain oxygenated compounds and terpenoids are jointly responsible for the distinctive odor differences between the two origins. Specifically, compounds such as linalool, carvone, and (E)-caryophyllene contribute significantly to the characteristic odor profiles of MCZ and BCZ.

This study has certain limitations. First, the sample size was relatively small (six batches per origin), and more samples from different geographical regions are needed for validation. Second, the relationship between these chemical differences and pharmacological effects requires further investigation. Third, the discriminatory efficiency of the established fingerprinting method needs validation with more authentic reference samples. Fourth, since MCZ and BCZ were collected from different geographic regions, the observed chemical differences, including the identified key volatile markers, may be confounded by geographic origin and cultivation conditions rather than solely reflecting species differences. Environmental factors such as temperature, light, soil properties, and water availability are known to significantly influence secondary metabolite accumulation in medicinal plants [62,63,64], and substantial geographic variation in essential oil composition has been documented across different *Atractylodes* cultivation regions [65,66]. Controlled cultivation experiments with both species grown under identical environmental conditions would be necessary to confirm whether these markers are truly species-specific. The 2CV results further validated the reliability of the OPLS-DA models, indicating that the discrimination between BCZ and MCZ was not an artifact of overfitting but reflected genuine metabolic and sensory differences between the two species.

Future studies should expand sample collection to include batches from different cultivation conditions and harvest times. In addition, metabolomics combined with pharmacological experiments may further elucidate the relationship between chemical constituents and therapeutic effects.

## 4. Materials and Methods

### 4.1. Reagents

Optima-grade methanol and acetonitrile were purchased from Thermo Fisher Scientific (Waltham, MA, USA). Purified water was obtained from Guangzhou Watsons Food & Beverage Co., Ltd. (Guangzhou, China). All solvents were used without further purification.

### 4.2. Herbal Materials

**Figure 14:**
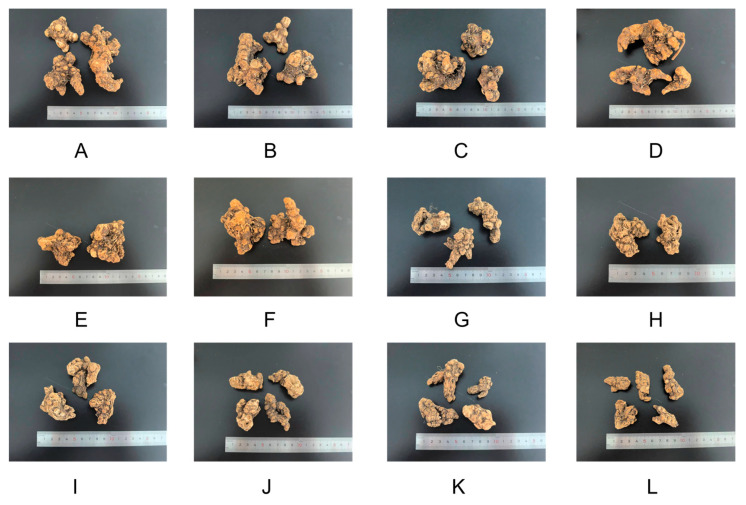
(**A**–**F**) are MCZ1–MCZ6; (**G**–**L**) are BCZ1–BCZ6.

A total of twelve batches of Atractylodis Rhizoma samples were used in this study, including six batches of MCZ and six batches of BCZ. The appearance characteristics of the herbal materials are shown in Figure 14, and the sample codes along with their geographical origins are provided in Table 4.

The MCZ samples were derived from *Atractylodes lancea* (Thunb.) DC., while the BCZ samples were derived from *Atractylodes chinensis* (DC.) Koidz. All samples were authenticated by Professor Fang Zhang from Shandong University of Traditional Chinese Medicine.

### 4.3. HS-GC-IMS Analysis

Analysis was performed using the FlavourSpec^®^ GC-IMS (G.A.S., Dortmund, Germany). Precisely weigh 0.5 g each of powdered MCZ and BCZ, place the samples into 20 mL headspace vials with stoppers, and incubate at 80 °C for 15 min prior to injection. The system parameters were as follows: column temperature, 60 °C; carrier gas, N2; ion mobility detector temperature, 45 °C; total analysis time, 40 min. Injection volume was 200 μL, incubation time 15 min, incubation temperature 80 °C, injection needle temperature 80 °C, and incubation speed 500 rpm. E1 drift gas flow rate was 75 mL/min. E2 carrier gas flow rates were: 0–2 min, 2 mL/min; 2–10 min, 2–10 mL/min; 10–20 min, 10–100 mL/min; 20–25 min, 100–150 mL/min; 25–40 min, 150 mL/min. Qualitative analysis was conducted using the system’s built-in software, NIST database, and IMS migration time database. Component fingerprint profiles were generated using the Reporter and Gallery Plot plugins. Each sample was analyzed in triplicate.

### 4.4. UPLC-Orbitrap Analysis

#### 4.4.1. Extraction of Non-Volatile Compounds

Pass the powders of MCZ and BCZ through a 60-mesh sieve. Accurately weigh 0.5 g of each sample, add exactly 20 mL of methanol, and weigh the mixture again to record its mass. Sonicate the mixture for 30 min, allow it to cool, and then reweigh. Add methanol to compensate for any weight loss, mix thoroughly, and transfer the solution to a 25 mL volumetric flask. Adjust to volume with methanol, filter the solution through a 0.22 µm microporous membrane, and store the resulting test solution at 4 °C in the dark until analysis. Each sample was prepared and analyzed in triplicate.

#### 4.4.2. Instrumental Conditions

Analysis was performed using UPLC-Q-Exactive-Orbitrap-MS (Thermo Fisher Scientific, Waltham, MA, USA). Chromatographic conditions: Waters Acquity UPLC BEH C18 column (2.1 mm × 100 mm, 1.7 µm); mobile phase: A-water, B-acetonitrile, gradient elution; flow rate: 0.30 mL/min; column temperature: 30 °C; injection volume: 5 µL. The gradient elution program was as follows: 0–2 min, 5–30% B; 2–14 min, 30–60% B; 14–23 min, 60–70% B; 23–31 min, 70–95% B; 31–31.5 min, 95–5% B; 31.5–35 min, 5% B. MS conditions: Electrospray ionization (ESI) was performed in both positive and negative ion modes, scanning a mass range of *m*/*z* 50–1000. For negative ion mode, the optimized ESI parameters were as follows: capillary temperature, 350 °C; sheath gas (nitrogen) flow rate, 30 arb; auxiliary gas (nitrogen) flow rate, 10 arb; source voltage, 3.0 kV; capillary voltage, −35 V; tube lens voltage, −110 V. For positive ion mode: capillary voltage, 25 V; source voltage, 4.0 kV; tube lens voltage, 110 V; other parameters were the same as those for negative ion mode. The Orbitrap mass analyzer resolution was set to 30,000. The secondary mass spectrometer operated in data-dependent acquisition (DDA) mode with a normalized collision energy of 35%.

### 4.5. Electronic Nose Analysis

PEN3 electronic nose system (AIRSENSE Analytics GmbH, Schwerin, Germany) was used to analyze the odors of BCZ and MCZ. This system is equipped with 10 odor sensors (W1C, W5S, W3C, W6S, W5C, W1S, W1W, W2S, W2W, W3S). By evaluating each sensor’s sensitivity to various compound classes, the primary volatile odor characteristics of both *Atractylodes* species were identified. The response characteristics of the electronic nose sensors are listed in Table 5.

Accurately weigh 2.0 g of each BCZ and MCZ powder and transfer to headspace vials. Seal the vials with double-layer sealing membranes and allow them to equilibrate at room temperature for 20 min before analysis. The headspace inhalation method was used, with stable response values serving as the evaluation criterion. Sampling was performed every 1 s, with a cleaning time of 120 s, zero adjustment for 5 s, pre-sampling for 5 s, and a sampling duration of 120 s. The injection flow rate was set at 600 mL/min. For each batch, three samples were weighed and analyzed in triplicate.

### 4.6. Electronic Tongue Analysis

An SA-402B electronic tongue (Insent Intelligent Sensor Technology Inc., Atsugi, Japan) was used to analyze samples of BCZ and MCZ. The samples were ground and passed through a No. 4 sieve (65 mesh). Accurately weigh 1.0 g of the powdered sample and place it in a 250 mL stoppered conical flask. Add 150 mL of deionized water, shake well, and sonicate at 100 W for 30 min. Transfer the entire extract to a centrifuge tube and centrifuge at 3000 rpm for 20 min. Collect 35 mL of the supernatant and place it in a dedicated electronic tongue sample cup for analysis. The raw data were converted into taste values using the instrument’s built-in data processing software and exported for further analysis. Detailed information on the electronic tongue sensors is provided in Table 6.

### 4.7. Software and Statistical Analysis

Qualitative analysis of volatile components and construction of fingerprint profiles were carried out using the built-in database and plugins of the FlavourSpec^®^ GC-IMS instrument. Principal Component Analysis (PCA) and Orthogonal Partial Least Squares Discriminant Analysis (OPLS-DA) were performed with SIMCA 14.1 software, and Variable Importance in Projection (VIP) values were calculated. Prism 10.1.2 was used to plot HS-GC-IMS component analysis charts; WinMuster software (AIRSENSE Analytics GmbH, Schwerin, Germany) was used to analyze electronic nose loadings plots; radar charts and stacked bar charts were created using Origin 2019b; and “component-odor” Pearson correlation analysis was conducted via the Microbioinformatics online platform. 2CV was performed using the pls package (version 2.9-0) in R (version 4.5.0). The outer loop employed leave-one-out cross-validation to assess prediction performance, while the inner loop utilized 7-fold cross-validation to optimize the number of orthogonal and predictive components. The 2CV Q^2^ was calculated based on the predicted versus observed Y values across all 12 iterations. Permutation tests (100 random permutations) were conducted to confirm the absence of overfitting.

## 5. Conclusions

In this study, a multi-dimensional analytical strategy integrating HS-GC-IMS, UPLC-Orbitrap MS, electronic nose, and electronic tongue was established to systematically characterize the differences between MCZ and BCZ.

HS-GC-IMS detected 108 volatile organic compounds, from which 24 volatile markers with VIP > 1.2 were screened as key contributors to the differentiation of MCZ and BCZ. Among them, propionic acid and 5-methyl-2-furancarboxaldehyde were mainly distributed in MCZ, whereas (E)-caryophyllene was present only or at higher levels in BCZ, indicating its potential as a characteristic volatile marker of BCZ. UPLC-Orbitrap MS detected 78 non-volatile constituents, and OPLS-DA screened 17 key non-volatile differential metabolites with VIP > 1.2, providing complementary chemical evidence for distinguishing the two origins. Electronic nose results revealed distinct odor profiles between MCZ and BCZ. Sensors W5S, W1W, and W1S were identified as the main differential sensors, suggesting that nitrogen oxides, terpenoids, inorganic sulfides, and short-chain alkanes are the primary contributors to the odor differences. Electronic tongue analysis demonstrated clear taste discrimination between the two origins, with sourness and richness identified as the key taste indicators.

Taken together, MCZ and BCZ can be clearly separated not only by sensory signals but also by both volatile and non-volatile chemical profiles. The integration of these four techniques provides a comprehensive and reliable strategy for distinguishing the two botanical origins of Atractylodis Rhizoma, offering scientific support for accurate authentication, quality evaluation, and rational clinical application. Future studies should expand sample collection to include batches from different geographic regions and cultivation conditions to further validate these markers.

## Figures and Tables

**Table 1:** Identification of volatile metabolites in *Atractylodes* of different origins using gas-phase ion mobility spectrometry.

Class	Compound	CAS	Formula	MW	RI	Rt [sec]	Dt [a.u.]	Differential Components
Alcohol	Geraniol	106-24-1	C_10_H_18_O	154.3	1837.9	1136.69	1.21	
	(Z)-4-Decen-1-ol	57074-37-0	C_10_H_20_O	156.3	1786.6	1061.73	1.44	
	Propylene glycol	57-55-6	C_3_H_8_O_2_	76.1	1586.9	814.13	1.27	
	Cedrol	77-53-2	C_15_H_26_O	222.4	1555.7	780.98	1.43	
	Linalool oxide	1365-19-1	C_10_H_18_O_2_	170.3	1465.3	692.55	1.67	
	2-Hexen-1-ol	2305-21-7	C_6_H_12_O	100.2	1395.0	628.38	1.19	
	(E)-3-Hexen-1-ol	928-97-2	C_6_H_12_O	100.2	1350.9	542.58	1.25	C5
	α-Terpineol	98-55-5	C_10_H_18_O	154.3	1283.5	433.41	1.22	C8
	2-Butyl-1-octanol	3913-02-8	C_12_H_26_O	186.3	1277.2	424.23	1.67	
	4-Methoxybenzyl alcohol	105-13-5	C_8_H_10_O_2_	138.2	1244.7	380.09	1.08	C11
	cis-3,7-Dimethyl-octa-2,6-dien-1-ol-M(FCC)	624-15-7	C_10_H_18_O	154.3	1219.4	349.03	1.22	
	FCC dimer	624-15-7	C_10_H_18_O	154.3	1217.2	346.37	1.64	C1
	3,7-Dimethyl-1-octanol	106-21-8	C_10_H_22_O	158.3	1200.1	327.03	1.57	
	(–)-4-Terpineol	20126-76-5	C_10_H_18_O	154.3	1178.5	304.86	1.22	
	1-Penten-3-ol	616-25-1	C_5_H_10_O	86.1	1155.0	282.94	0.94	
	2-Pentanol	6032-29-7	C_5_H_12_O	88.1	1153.9	281.93	1.44	C21
	β-Linalool	78-70-6	C_10_H_18_O	154.3	1098.7	236.61	1.21	
	1,8-Cineole	470-82-6	C_10_H_18_O	154.3	1027.2	203.95	1.29	
	2-Hexanol	626-93-7	C_6_H_14_O	102.2	966.5	179.99	1.28	
	Isopropyl alcohol	67-63-0	C_3_H_8_O	60.1	942.9	171.44	1.09	
	2-Butoxyethanol	111-76-2	C_6_H_14_O_2_	118.2	895.0	155.34	1.20	
Aldehyde	(E)-2-Decenal	3913-81-3	C_10_H_18_O	154.3	1635.7	868.64	1.47	
	(E,Z)-2,6-Nonadienal	557-48-2	C_9_H_14_O	138.2	1583.7	810.64	1.37	
	5-Methylfurfural	620-02-0	C_6_H_6_O_2_	110.1	1555.7	780.97	1.13	
	Undecanal	112-44-7	C_11_H_22_O	170.3	1316.3	483.49	1.594	
	3,7-Dimethyl-2,6-octadienal	5392-40-5	C_10_H_16_O	152.2	1267.6	410.70	1.042	
	Cuminaldehyde	122-03-2	C_10_H_12_O	148.2	1242.5	377.25	1.330	
	(E)-2-Hexenal	6728-26-3	C_6_H_10_O	98.1	1207.2	334.86	1.173	C18
	4-Ethylbenzaldehyde	4748-78-1	C_9_H_10_O	134.2	1199.9	326.74	1.271	
	(E)-2-Octenal	2548-87-0	C_8_H_14_O	126.2	1024.5	202.82	1.328	
	3-Methylbutanal	590-86-3	C_5_H_10_O	86.1	921.3	164.00	1.194	C6
	(E,E)-2,4-Hexadienal	142-83-6	C_6_H_8_O	96.1	907.6	159.44	1.114	
	(Z)-4-Heptenal	6728-31-0	C_7_H_12_O	112.2	881.9	151.20	1.145	
	Furfural	98-01-1	C_5_H_4_O_2_	96.1	845.2	140.21	1.089	
Ketone	2,4-Dimethylcyclopentan-1,3-dione	13494-06-9	C_7_H_10_O_2_	126.2	1900.2	1234.98	1.204	
	2-Hydroxy-3-methyl-2-cyclopenten-1-one	80-71-7	C_6_H_8_O_2_	112.1	1834.6	1131.77	1.520	C22
	3-Methyl-2(5H)-furanone	22122-36-7	C_5_H_6_O_2_	98.1	1798.0	1078.04	1.381	
	3-Methyl-2-cyclopenten-1-one	2758-18-1	C_6_H_8_O	96.1	1516.2	741.06	1.109	
	2-Nonanone	821-55-6	C_9_H_18_O	142.2	1414.0	646.87	1.405	
	6-Methyl-5-hepten-2-one	110-93-0	C_8_H_14_O	126.2	1335.2	514.89	1.184	
	Hydroxyacetone	116-09-6	C_3_H_6_O_2_	74.1	1299.0	456.56	1.043	
	Carvone (monomer)	2244-16-8	C_10_H_14_O	150.2	1261.2	401.89	1.303	C2
	Carvone (dimer)	2244-16-8	C_10_H_14_O	150.2	1260.1	400.37	1.831	
	2-Hexanone	591-78-6	C_6_H_12_O	100.2	1106.3	242.39	1.191	
	2,3-Pentanedione	1629-58-9	C_5_H_8_O	84.1	1005.0	194.83	1.075	
	2-Octanone	111-13-7	C_8_H_16_O	128.2	982.5	186.01	1.335	C24
	2-Butanone	78-93-3	C_4_H_8_O	72.1	941.1	170.82	1.059	C13
	Cyclopentanone	120-92-3	C_5_H_8_O	84.1	810.1	130.42	1.102	
Carboxylic acid	(E)-3-Hexenoic acid	1577-18-0	C_6_H_10_O_2_	114.1	1955.5	1329.31	1.229	C20
	Heptanoic acid	111-14-8	C_7_H_14_O_2_	130.2	1918.8	1265.87	1.369	
	Propanoic acid	79-09-4	C_3_H_6_O_2_	74.1	1491.6	717.12	1.105	
	Hexanoic acid	142-62-1	C_6_H_12_O_2_	116.2	1001.0	193.22	1.293	
Ester	γ-Decalactone	706-14-9	C_10_H_18_O_2_	170.3	2119.5	1653.17	1.494	
	Benzyl phenylacetate	140-11-4	C_16_H_16_O_2_	240.3	1713.1	962.89	1.326	
	Citronellyl formate	105-85-1	C_11_H_20_O_2_	184.3	1615.7	845.86	1.335	
	Furfuryl acetate	623-17-6	C_7_H_8_O_3_	140.1	1535.5	760.28	1.411	C9
	Propylene glycol diacetate	623-84-7	C_7_H_12_O_4_	160.2	1487.6	713.36	1.216	
	Ethyl 4-methoxybenzoate	94-30-4	C_10_H_12_O_3_	180.2	1405.9	639.93	1.331	
	Benzyl isobutyrate (dimer)	103-48-0	C_12_H_16_O_2_	192.3	1390.6	619.09	1.976	
	Benzyl isobutyrate (monomer)	103-48-0	C_12_H_16_O_2_	192.3	1390.0	617.96	1.393	
	Linalyl isobutyrate (monomer)	78-35-3	C_14_H_24_O_2_	224.3	1355.0	549.96	1.218	C3
	Linalyl isobutyrate (dimer)	78-35-3	C_14_H_24_O_2_	224.3	1351.9	544.34	1.696	
	Ethyl 2-hydroxypropanoate	97-64-3	C_5_H_10_O_3_	118.1	1337.1	518.22	1.133	
	Methyl anthranilate	134-20-3	C_8_H_9_NO_2_	151.2	1297.5	454.17	1.264	C7
	Methyl heptanoate	106-73-0	C_8_H_16_O_2_	144.2	1297.1	453.62	1.360	
	Linalyl acetate	115-95-7	C_12_H_20_O_2_	196.3	1260.9	401.50	1.212	
	β-Methyl-γ-octalactone	39212-23-2	C_9_H_16_O_2_	156.2	1201.2	328.21	1.371	
	1-Phenylethyl acetate	93-92-5	C_10_H_12_O_2_	164.2	1192.4	318.60	1.049	C14
	Methyl phenylacetate	101-41-7	C_9_H_10_O_2_	150.2	1154.0	282.03	1.255	
	2-Methylbutyl acetate	624-41-9	C_7_H_14_O_2_	130.2	1128.7	260.22	1.292	
	Tetrahydrofurfuryl acetate	637-64-9	C_7_H_12_O_3_	144.2	1099.1	236.91	1.180	
	3-Methylbutyl 2-methylbutanoate	27625-35-0	C_10_H_20_O_2_	172.3	1098.6	236.52	1.423	
	γ-Hexalactone	695-06-7	C_6_H_10_O_2_	114.1	1064.6	220.29	1.185	
	Prenyl acetate	1191-16-8	C_7_H_12_O_2_	128.2	932.5	167.82	0.944	
	Butyl propionate	590-01-2	C_7_H_14_O_2_	130.2	893.6	154.88	1.282	
Alkene	(E,E)-α-Farnesene	502-61-4	C_15_H_24_	204.4	1735.7	992.26	1.435	C10
	(E)-Caryophyllene	87-44-5	C_15_H_24_	204.4	1383.1	603.82	1.424	C19
	Limonene	138-86-3	C_10_H_16_	136.2	1233.6	366.14	1.218	
	Naphthalene	91-20-3	C_10_H_8_	128.2	1155.6	283.44	1.113	
	δ-3-Carene	13466-78-9	C_10_H_16_	136.2	1143.1	272.39	1.216	C16
	α-Phellandrene	99-83-2	C_10_H_16_	136.2	1014.0	198.49	1.221	
Phenol	Methyl eugenol	93-15-2	C_11_H_14_O_2_	178.2	2040.9	1489.05	1.443	C15
	Butylated hydroxytoluene (monomer)	128-37-0	C_15_H_24_O	220.4	1517.8	742.64	1.329	
	Butylated hydroxytoluene (dimer)	128-37-0	C_15_H_24_O	220.4	1496.9	722.25	1.331	C17
	Ethyl vanillin	121-32-4	C_9_H_10_O_3_	166.2	1428.7	659.58	1.278	
	4-vinyl-2-methoxyphenol (monomer)	7786-61-0	C_9_H_10_O_2_	150.2	1319.6	488.95	1.218	
	4-vinyl-2-methoxyphenol (dimer)	7786-61-0	C_9_H_10_O_2_	150.2	1319.2	488.22	1.732	
	4-Vinylguaiacol	7786-61-0	C_9_H_10_O_2_	150.2	1290.7	443.97	1.218	C12
	Methyl salicylate	119-36-8	C_8_H_8_O_3_	152.1	1163.4	290.54	1.204	
N-containing	2-Methoxy-3-sec-butylpyrazine	24168-70-5	C_9_H_14_N_2_O	166.2	1516.4	741.24	1.249	C23
	2-Ethyl-3-methylpyrazine	15707-23-0	C_7_H_10_N_2_	122.2	1434.0	664.25	1.161	
	Pyrazine	290-37-9	C_4_H_4_N_2_	80.1	1211.5	339.85	1.044	
	N-Ethyl-2-pyrrolidone	2687-91-4	C_6_H_11_NO	113.2	1152.9	281.06	1.154	
	N,N-Dimethylaniline	121-69-7	C_9_H_13_N	135.2	1015.9	199.24	1.225	
	2-Ethylpyridine	100-71-0	C_7_H_9_N	107.2	969.1	180.94	1.098	
	1-Methyl-1H-pyrrole-2-carboxaldehyde	1192-58-1	C_6_H_7_NO	109.1	966.1	179.85	1.121	
S-containing	5-Methyl-2-thiophenecarboxaldehyde	13679-70-4	C_6_H_6_OS	126.2	1129.1	260.60	1.179	
	Ethyl thioacetate	625-60-5	C_4_H_8_OS	104.2	1117.4	251.12	1.131	
	2,5-Dimethylthiophene	638-02-8	C_6_H_8_S	112.2	885.6	152.36	1.070	
	Dimethyl disulfide	624-92-0	C_2_H_6_S_2_	94.2	737.7	112.35	1.140	
Other	N,N-Dimethylacetamide	127-19-5	C_4_H_9_NO	87.1	1434.2	664.43	1.063	
	N,N-Dimethylformamide	68-12-2	C_3_H_7_NO	73.1	1372.6	583.21	1.252	C4
	Dihydrocoumarin	119-84-6	C_9_H_8_O_2_	148.2	1352.3	545.04	1.287	
	Anethole	4180-23-8	C_10_H_12_O	148.2	1288.2	440.29	1.761	
	Triethyl phosphate	78-40-0	C_6_H_15_O_4_P	182.2	1138.1	268.17	1.299	
	Octamethylcyclotetrasiloxane	556-67-2	C_8_H_24_O_4_Si_4_	296.6	1004.1	194.49	1.676	
	2-Pentylfuran	3777-69-3	C_9_H_14_O	138.2	1001.7	193.51	1.246	
	4-Methylanisole	104-93-8	C_8_H_10_O	122.2	993.5	190.29	1.116	

**Table 2:** Chemical composition of non-volatile metabolites in *Atractylodes*.

Class	Name	Formula	DeltaMass [ppm]	MW	*m*/*z*	RT [min]	Fragment Ions
Flavonoids	Nobiletin	C_21_H_22_O_8_	−1.67	402.13	403.14	16.27	403.14, 388.11
	Daidzein	C_15_H_10_O_4_	−1.22	254.06	255.06	9.64	255.06, 227.07
Lignans	Matairesinol	C_20_H_22_O_6_	−1.46	358.14	359.15	9.64	323.12, 291.1
	(3R,4R)-4-[(3,4-dimethoxyphenyl)methyl]-3-[(4-hydroxy-3-methoxyphenyl)methyl]oxolan-2-one	C_21_H_24_O_6_	−1.49	372.16	373.16	11.39	306.12, 237.11
Others	2,4-di-tert-Butylphenol	C_14_H_22_O	−0.3	206.17	205.16	26.42	206.16, 205.16
	trans-3-Indoleacrylic acid	C_11_H_9_NO_2_	−1.81	187.06	188.07	3.1	188.07, 170.07
	1,2,3,4-Tetramethyl-1,3-cyclopentadiene	C_9_H_14_	−2	122.11	123.12	26.99	123.12, 95.09
	(±)-Cannabichromeorcin	C_17_H_22_O_2_	−0.84	258.16	259.17	14.8	259.17, 203.11
Sugars and their glycosides	Gluconic acid	C_6_H_12_O_7_	−0.79	196.06	195.05	1.07	195.05, 177.04
	α,α-Trehalose	C_12_H_22_O_11_	−0.79	342.12	341.11	1.13	341.11, 179.06
	Puerarin	C_21_H_20_O_9_	−0.84	416.11	417.12	4.17	381.1, 363.09
	3-Ethyl-4-hydroxy-4-methylpentyl 6-O-[(2S,3R,4R)-3,4-dihydroxy-4-(hydroxymethyl)tetrahydro-2-furanyl]-β-D-glucopyranoside	C_19_H_36_O_11_	−0.17	440.23	439.22	5.42	307.18, 161.05
	5′-S-Methyl-5′-thioadenosine	C_11_H_15_N_5_O_3_S	−1.44	297.09	298.1	4.32	136.06, 61.01
	D-(−)-Fructose	C_6_H_12_O_6_	−0.94	180.06	179.06	1.08	179.07, 161.05
	2′-O-Methyladenosine	C_11_H_15_N_5_O_4_	−1.71	281.11	282.12	3.23	283.17, 282.12
	8-Hydroxy-6-methoxy-2-oxo-2H-chromen-7-yl β-D-glucopyranoside	C_16_H_18_O_10_	−0.37	370.09	369.08	3.8	354.06, 207.03
Terpenoids	Nootkatone	C_15_H_22_O	−2.18	218.17	219.17	12.51	219.17, 163.11
	(−)-Caryophyllene oxide	C_15_H_24_O	−1.48	220.18	221.19	17.97	221.19, 203.18
	(3aS,5aR,6R,9aS,9bS)-6-hydroxy-5a-methyl-3,9-dimethylidene-dodecahydronaphtho[1,2-b]furan-2-one	C_15_H_20_O_3_	−1.45	248.14	249.15	10.71	249.15, 231.14
	(3aR,5aR,9bR)-3a-hydroxy-5a,9-dimethyl-3-methylidene-2H,3H,3aH,4H,5H,5aH,6H,7H,8H,9bH-naphtho[1,2-b]furan-2-one	C_15_H_20_O_3_	−1.06	248.14	249.15	14.52	249.15, 231.14
	(3aR,4aS,5R,7aS,8S,9aR)-5-Hydroxy-4a,8-dimethyl-3-methyleneoctahydroazuleno[6,5-b]furan-2,6(3H,4H)-dione	C_15_H_20_O_4_	−1.57	264.14	265.14	4.8	265.14, 229.12
	(3aR,7aS,8S,9aR)-5,8-dimethyl-3-methylidene-2H,3H,3aH,4H,6H,7H,7aH,8H,9H,9aH-azuleno[6,5-b]furan-2,6-dione	C_15_H_18_O_3_	−1.18	246.13	247.13	12.69	247.13, 229.12
	Ambrosic acid	C_15_H_20_O_4_	−1.54	264.14	265.14	7.37	265.14, 229.12
	6-hydroxy-3,5a,9-trimethyl-2H,3H,3aH,4H,5H,5aH,6H,7H,9aH,9bH-naphtho[1,2-b]furan-2-one	C_15_H_22_O_3_	−1.51	250.16	251.16	11.42	233.15, 187.15
	Zedoarondiol	C_15_H_24_O_3_	−1.38	252.17	253.18	9.47	235.17, 217.16
	D,L-Camphor	C_10_H_16_O	−1.69	152.12	153.13	8.29	153.13, 135.09
	(5E)-7-methylidene-10-oxo-4-(propan-2-yl)undec-5-enoic acid	C_15_H_24_O_3_	−1.41	252.17	253.18	8.34	235.17, 217.16
	Atractylone	C_15_H_20_O	−1.74	216.15	217.16	18.62	217.16, 175.15
	Atractylenolide IV	C_17_H_22_O_5_	−2.12	306.15	307.15	16.31	290.15, 215.11
	Atractylodinol	C_13_H_10_O_2_	−3.01	198.08	199.07	19.06	290.15, 215.11
	Muscone	C_16_H_30_O	−1.86	238.23	239.24	28.75	239.24, 151.15
Amides	Hexadecanamide	C_16_H_33_NO	−2.31	255.26	256.26	31.62	257.27, 116.11
	Oleamide	C_18_H_35_NO	−1.7	281.27	282.28	28.03	282.28, 149.13
	Stearamide	C_18_H_37_NO	−0.94	283.29	284.29	33.11	284.29, 172.17
	Erucamide	C_22_H_43_NO	−1.98	337.33	338.34	31.59	338.34, 303.31
	Linoleoyl ethanolamide	C_20_H_37_NO_2_	−2.06	323.28	324.29	30.17	306.28, 161.13
Coumarins	Scopoletin	C_10_H_8_O_4_	−1.48	192.04	191.03	5.16	193.05, 178.03
	Scoparone	C_11_H_10_O_4_	−2.15	206.06	207.06	6.43	207.06, 192.04
	4-Hydroxycoumarin	C_9_H_6_O_3_	−0.72	162.03	161.02	5.23	161.02, 162.03
	Fraxetin	C_10_H_8_O_5_	−1.44	208.04	209.04	4.52	209.04, 194.02
Organic acids and their esters	DL-Malic acid	C_4_H_6_O_5_	−0.55	134.02	133.01	1.44	133.01, 115
	DL-Arginine	C_6_H_14_N_4_O_2_	−1.8	174.11	175.12	1.66	175.12, 158.09
	D-(+)-Tryptophan	C_11_H_12_N_2_O_2_	−0.65	204.09	203.08	3.1	203.08, 159.09
	Proline	C_5_H_9_NO_2_	−2.36	115.06	116.07	1.09	116.07, 117.07
	Isoleucine	C_6_H_13_NO_2_	−1.74	131.09	132.1	1.53	132.1, 87.09
	Salicylic acid	C_7_H_6_O_3_	−0.46	138.03	137.02	3.51	138.03, 137.02
	4-Feruloylquinic acid	C_17_H_20_O_9_	−0.1	368.11	367.1	3.35	193.05, 173.05
	Benzoic acid	C_7_H_6_O_2_	−0.27	122.04	121.03	3.46	121.03, 122.02
	D-(−)-Quinic acid	C_7_H_12_O_6_	−0.37	192.06	191.06	2.37	191.06, 173.05
	α-Eleostearic acid	C_18_H_30_O_2_	−2.22	278.22	279.23	27.23	279.23, 173.13
	4,5-Dicaffeoylquinic acid	C_25_H_24_O_12_	−0.1	516.13	515.12	4.96	353.1, 191.06
	Picolinic acid	C_6_H_5_NO_2_	−0.91	123.03	122.02	3.16	122.02, 94.03
	Neochlorogenic acid	C_16_H_18_O_9_	−0.32	354.09	353.09	4.71	191.06, 179.03
	Chlorogenic acid	C_16_H_18_O_9_	−0.31	354.09	353.09	2.48	191.06, 179.04
	3-Furoic acid	C_5_H_4_O_3_	−0.81	112.02	111.01	1.23	111.01, 67.02
	Catechol	C_6_H_6_O_2_	−0.44	110.04	109.03	3.46	109.03, 108.02
	Pentadecanoic acid	C_15_H_30_O_2_	−0.64	242.22	241.22	31.68	241.22, 129.23
	16-Hydroxyhexadecanoic acid	C_16_H_32_O_3_	−0.44	272.24	271.23	27.1	271.23, 235.22
	(+/−)9,10-dihydroxy-12Z-octadecenoic acid	C_18_H_34_O_4_	0.1	314.25	313.24	24.35	313.24, 295.23
	Caffeic acid	C_9_H_8_O_4_	−0.33	180.04	179.03	2.88	179.03, 135.05
	Nervonic acid	C_24_H_46_O_2_	−0.74	366.35	365.34	32.28	365.34, 366.35
	9-Oxo-10(E),12(E)-octadecadienoic acid	C_18_H_30_O_3_	−0.86	294.22	295.23	26.65	277.22, 165.13
	Vanillic acid	C_8_H_8_O_4_	−0.52	168.04	167.03	2.71	167.03, 152.01
	Syringic acid	C_9_H_10_O_5_	−0.54	198.05	197.05	2.55	197.05, 182.02
	(±)13-HODE	C_18_H_32_O_3_	−0.91	296.23	295.23	28.09	295.23, 277.22
	8Z,11Z,14Z-Eicosatrienoic acid	C_20_H_34_O_2_	−0.38	306.26	305.25	32	305.25, 306.35
	Corchorifatty acid F	C_18_H_32_O_5_	0.38	328.23	327.22	14.56	327.22, 239.13
	(±)9-HpODE	C_18_H_32_O_4_	0.12	312.23	311.22	23.23	311.22, 293.21
	1-Linoleoyl glycerol	C_21_H_38_O_4_	−1.87	354.28	355.28	30.81	285.01, 263.24
	13(S)-HOTrE	C_18_H_30_O_3_	−0.81	294.22	295.23	23.18	277.22, 165.13
	Methyl salicylate	C_8_H_8_O_3_	−0.35	152.05	151.04	6.94	151.04, 136.02
	Citric acid	C_6_H_8_O_7_	−0.55	192.03	191.02	1.44	191.06, 129.02
	2-Methylbenzoic acid	C_8_H_8_O_2_	−0.57	136.05	135.05	6.6	136.05, 135.05
	3-Hydroxypicolinic acid	C_6_H_5_NO_3_	−1.44	139.03	140.03	4.26	183.08, 165.07
	(15Z)-9,12,13-Trihydroxy-15-octadecenoic acid	C_18_H_34_O_5_	−0.22	330.24	329.23	16.01	329.23, 311.22
	trans-10-Heptadecenoic acid	C_17_H_32_O_2_	−0.05	268.24	267.23	31.95	267.23, 249.22
	Methyl cinnamate	C_10_H_10_O_2_	−2.18	162.07	163.08	15.91	163.07, 131.05
	9-Oxo-ODE	C_18_H_30_O_3_	−1.03	294.22	295.23	28.67	277.22, 165.13

**Table 3:** Double cross-validation (2CV) results for OPLS-DA models.

Model	Q^2^ (2CV)	Permutation Q^2^ Intercept	Accuracy
Electronic nose	0.624	−1.680	91.67%
Electronic tongue	0.996	−1.621	100%
HS-GC-IMS	0.880	−2.281	100%
UPLC-Orbitrap MS	0.878	−1.269	100%

**Table 4:** Herbal material information table.

No.	Origin	No.	Origin
MCZ-1	Yingshan County, Hubei Province	BCZ-1	Chifeng City, Inner Mongolia Autonomous Region
MCZ-2	Yingshan County, Hubei Province	BCZ-2	Chifeng City, Inner Mongolia Autonomous Region
MCZ-3	Yingshan County, Hubei Province	BCZ-3	Hulunbuir City, Inner Mongolia Autonomous Region
MCZ-4	Shiyan City, Hubei Province	BCZ-4	Hulunbuir City, Inner Mongolia Autonomous Region
MCZ-5	Shiyan City, Hubei Province	BCZ-5	Chengde City, Hebei Province
MCZ-6	Shiyan City, Hubei Province	BCZ-6	Chengde City, Hebei Province

**Table 5:** Electronic nose sensor information table.

Serial Number	Sensor Name	Representative Compound Categories
R (1)	W1C	Sensitive to aromatic compounds and benzene compounds
R (2)	W5S	High sensitivity, highly responsive to nitrogen oxides
R (3)	W3C	Sensitive to amines and aromatic compounds
R (4)	W6S	Primarily selective for hydrides
R (5)	W5C	Alkane and aromatic components
R (6)	W1S	Sensitive to methane and other short-chain alkanes
R (7)	W1W	Sensitive to terpenes and inorganic sulfides
R (8)	W2S	Sensitive to alcohols, aldehydes, and ketones
R (9)	W2W	Aromatic compounds, sensitive to organic sulfides
R (10)	W3S	Sensitive to alkanes, long-chain alkanes

**Table 6:** Information on electronic tongue sensors.

Sensor	Taste	Aftertaste
Umami (AAE)	Umami	Richness
Salty (CTO)	Salty	-
Acidity (CAO)	Acidity	-
Bitterness (COO)	Bitterness	Bitter aftertaste
Astringency (AE1)	Astringency	Astringent aftertaste

## Data Availability

The data presented in this study are available on request from the corresponding author.
